# Immunotherapy for targeting cancer stem cells in hepatocellular carcinoma

**DOI:** 10.7150/thno.54648

**Published:** 2021-01-19

**Authors:** Xiaomeng Dai, Yixuan Guo, Yan Hu, Xuanwen Bao, Xudong Zhu, Qihan Fu, Hangyu Zhang, Zhou Tong, Lulu Liu, Yi Zheng, Peng Zhao, Weijia Fang

**Affiliations:** Department of Medical Oncology, The First Affiliated Hospital, College of Medicine, Zhejiang University, Hangzhou, China.

**Keywords:** hepatocellular carcinoma, cancer stem cells, immune evasion, targeting, immunotherapy

## Abstract

The rapid development and remarkable success of checkpoint inhibitors have provided significant breakthroughs in cancer treatment, including hepatocellular carcinoma (HCC). However, only 15-20% of HCC patients can benefit from checkpoint inhibitors. Cancer stem cells (CSCs) are responsible for recurrence, metastasis, and local and systemic therapy resistance in HCC. Accumulating evidence has suggested that HCC CSCs can create an immunosuppressive microenvironment through certain intrinsic and extrinsic mechanisms, resulting in immune evasion. Intrinsic evasion mechanisms mainly include activation of immune-related CSC signaling pathways, low-level expression of antigen presenting molecules, and high-level expression of immunosuppressive molecules. External evasion mechanisms are mainly related to HBV/HCV infection, alcoholic/nonalcoholic steatohepatitis, hypoxia stimulation, abnormal angiogenesis, and crosstalk between CSCs and immune cells. A better understanding of the complex mechanisms of CSCs involved in immune evasion will contribute to therapies for HCC. Here we will outline the detailed mechanisms of immune evasion for CSCs, and provide an overview of the current immunotherapies targeting CSCs in HCC.

## Introduction

Hepatocellular carcinoma (HCC) is one of the leading causes of cancer-associated deaths worldwide, accounting for approximately 75-85% of primary liver cancers 1, 2. Hepatitis B virus (HBV), hepatitis C virus (HCV), alcoholic, and nonalcoholic steatohepatitis (NASH) are major risk factors in the development of HCC 3. The tumor burden is highest in East Asia (more than 50% in China) and Africa because of HBV infection, while HCC incidence and mortality are increasing rapidly in the United States and Europe due to alcohol consumption and NASH 4. Most patients with HCC are diagnosed at advanced stages with liver disease and cirrhosis, missing the opportunity for surgery. Despite several advances in the treatment of HCC, particularly in targeted therapy and immunotherapy, the 5-year survival rate remains poor 5. Drug resistance, tumor metastasis and recurrence are the major causes of poor prognosis in HCC patients.

Cancer stem cells (CSCs) have been shown to be responsible for recurrence, metastasis, and local and systemic therapy resistance in HCC 6. Moreover, an overwhelming number of studies have suggested that CSCs can form an immunosuppressive microenvironment through both intrinsic and extrinsic mechanisms to induce ineffective antitumor immune responses 7. Application of immunotherapy, especially programmed cell death protein 1 (PD-1) and programmed cell death ligand 1 (PD-L1) monoclonal antibodies, to a variety of solid tumors (including HCC) represents a major breakthrough in cancer treatment 8, 9. However, most patients who have received immunotherapy still experience progression and metastasis 10. Considering that CSCs are a reservoir for the progression and metastasis of HCC, immunotherapy that targets CSCs may be an exciting research field.

**Figure 1:**
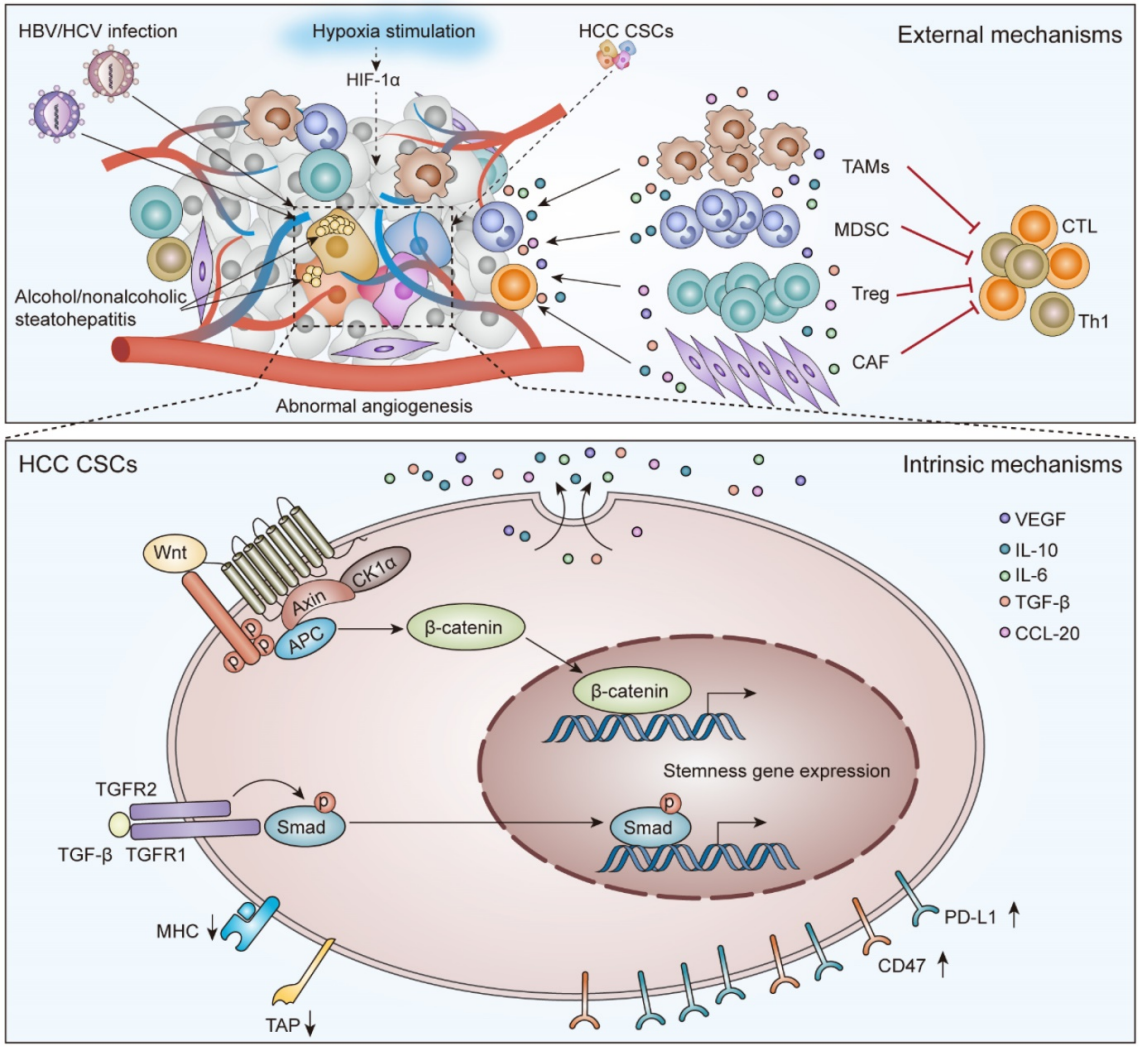
**The external and intrinsic mechanisms to mediate immunotherapy resistance for CSCs in HCC.** External evasion mechanisms are mainly related to HBV/HCV infection, alcohol/nonalcoholic steatohepatitis, hypoxia stimulation, abnormal angiogenesis, and crosstalk between CSCs and immune cells. Intrinsic evasion mechanisms mainly include activation of the Wnt/β-Catenin signaling pathway and TGF-β signaling pathway, low-level expression of TAP and/or MHC molecules, and high-level expression of CD47 and PD-L1.

**Figure 2:**
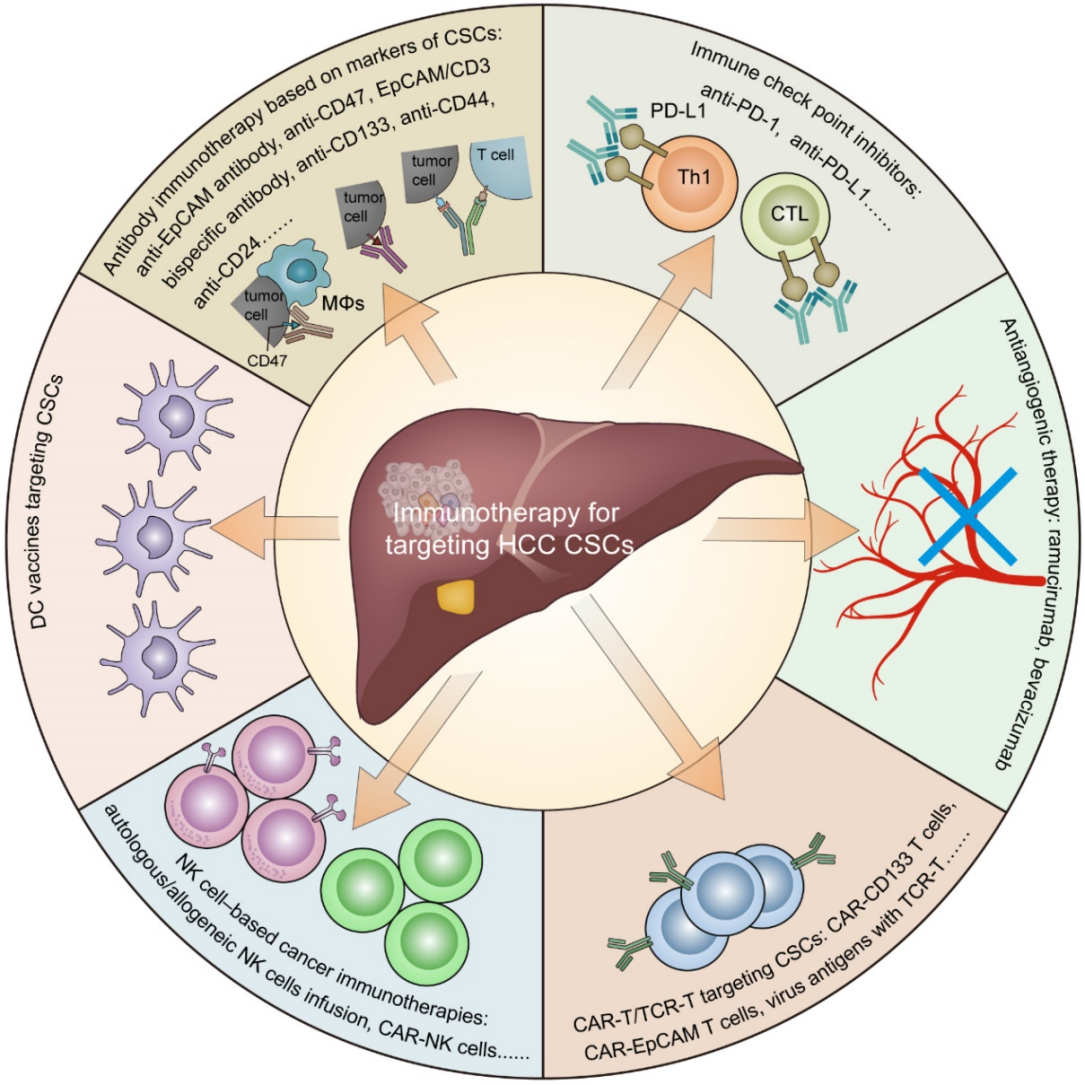
** Immunotherapy for targeting cancer stem cells in hepatocellular carcinoma.** Including antibody immunotherapy based on CSC markers, immune checkpoint inhibitors, antiangiogenic therapy, CAR-T/TCR-T cell therapy, NK cell-based cancer immunotherapies, and DC vaccines.

In this review, we summarize the role of CSCs in the tumor immunosuppressive environment (**Figure 1**) and provide an overview of the current immunotherapies targeting CSCs in HCC (**Figure 2**).

## CSCs and immune evasion in HCC

CSCs are a small population of cells that can self-renew and differentiate to initiate and maintain tumor growth 11. T Lapidot and colleagues first observed the existence of CSCs by demonstrating that CD34^+^/CD38^-^ myeloid leukemia (AML) cells have the ability to initiate tumors in NOD/SCID mice 12. HCC stem cells were first identified as side population (SP) cells by Haraguchi and colleagues in 2006 13, 14. They found that SP cells in HCC were more resistant to chemotherapy drugs (including 5-fluorouracil, doxorubicin and gemcitabine) than non-SP cells. Chiba *et al.* confirmed that as few as 1000 HCC SP cells have tumorigenic ability in NOD/SCID mice, whereas up to 1×10^6^ non-SP cells were unable to initiate tumors 15. Since then, according to xenotransplantation experiments, several cellular biomarkers of CSCs in HCC have been identified, including epithelial cell adhesion molecule (EpCAM), CD133, CD44, CD90, CD13, CD24, OV6, CD47, calcium channel α2δ1 isoform5, and intercellular adhesion molecule 1 (ICAM-1) 6, 16. Moreover, related studies showed that high expression of these CSC markers was associated with poor prognosis in HCC patients 17-22.

The existence of HCC CSCs indicates tumor heterogeneity and hierarchy, which is a hallmark feature of resistance to immunotherapy 23, 24. Zheng and colleagues observed that CSCs are also heterogeneous, as determined by single-cell transcriptome and functional analysis of HCC cells. They found that different CSC subpopulations have distinct molecular signatures that were independently correlated with poor prognosis in HCC patients 25. After decades of research, CSCs were found to mediate immunotherapy resistance through various intrinsic and external mechanisms 26. Intrinsic mechanisms of immune evasion include related stem cell pathway activation, the low-level expression of cellular antigen processing and presentation molecules, and the high-level expression of CD47 and PD-L1. External mechanisms of immune evasion include HBV/HCV infection, alcoholic/nonalcoholic steatohepatitis, hypoxia stimulation, abnormal angiogenesis, and infiltration of suppressive immune cells (**Figure 1**) 27-29.

## Intrinsic factors of immune evasion

### CSC signaling pathways and immune evasion

In HCC CSCs, signaling pathways involved in self-renewal and differentiation characteristics mainly include the Wnt/β-Catenin signaling pathway, Notch signaling pathway, Hedgehog signaling pathway, TGF-β signaling pathway, and AKT signaling pathway 26, 30, 31. The Wnt/β-Catenin signaling pathway and TGF-β signaling pathway are closely related to immune evasion in HCC 32. Intriguing studies have demonstrated that the aberrant activation of the tumor-intrinsic Wnt/β-Catenin signaling pathway correlates with a low proportion of T cell infiltration in the tumor microenvironment (TME) of HCC and melanoma tumor samples 33, 34. Tang and colleagues suggested that there was a functional link between the TGF-β signaling pathway and IL-6 in HCC 35. Moreover, IL-6 (Th2 cytokine) and TGF-β play an important role in the generation of an inhibitory immune microenvironment, antagonizing cytotoxic T lymphocytes (CTLs) and inducing antitumor immunity 36, 37. Other studies have found that Notch pathway activation was associated with low CTL activity by recruiting tumor-associated macrophages (TAMs) or myeloid-derived suppressor cells (MDSCs) in other tumors (including pancreatic cancer, ovarian cancer, prostate cancer) 38-41.

### Immunological properties of CSCs in HCC

A major mechanism by which CSCs avoid being attacked by the immune system involves minimization of antigenicity by downregulating key components of the cellular antigen processing and presentation machinery, mainly including transporters associated with antigen processing (TAP) and/or major histocompatibility complex (MHC) molecules 7, 32. CSCs or other tumor cells of HCC lack targetability due to rare presentation by human leukocyte antigen (HLA) complexes 42. In addition, Di Tomaso *et al*. found that glioblastoma CSCs were weakly positive and negative for MHC-I and MHC-II, leading to a lack of a T cell-mediated immune response 43. Interestingly, downregulation or loss of HLA-I/II expression in spheres was also observed in tumor spheres (including colon, pancreas, and breast carcinoma), which were composed of CSCs 44. Thus, downregulation or defects in antigen processing and presentation molecules provide a means for CSCs to evade CTL-mediated immune responses.

Additionally, CSCs have been found to express high levels of CD47 (the “don't eat me” signal), which inhibits macrophage phagocytosis by binding to its cognate ligand, signal-regulatory-protein-α (SIRPα) 45, 46. Lee and colleagues suggested that CD47 is preferentially expressed in liver CSCs, contributing to tumor initiation, self-renewal, and metastasis, and is significantly associated with poor clinical outcome 47. Therefore, CD47 has been identified as a marker of CSCs in HCC 16. Moreover, the high expression of CD47 in sorafenib-resistant HCC cells and samples is dependent on NF-κB expression 48. TAM-derived IL-6 induced CD47 upregulation in HCC through activation of the STAT3 pathway and correlated with poor survival in HCC patients 49. In summary, CSCs with high expression of CD47 in HCC can effectively avoid phagocytosis by macrophages and thus provide CSCs with a means of immune evasion.

Moreover, accumulating evidence has indicated that CSCs express high levels of PD-L1, which induce T cell apoptosis by binding to its cognate receptor PD-1. Hsu *et al*. demonstrated that epithelial-mesenchymal transition (EMT) enriched more PD-L1 in CSCs of breast and colon cancer cells by the EMT/β-catenin/STT3/PD-L1 signaling axis than non-CSCs 50. In the case of squamous cell carcinoma of the head and neck (SCCHN), PD-L1 was also highly expressed on CD44^+^ cells (CSCs) compared to CD44^-^ cells (non-CSCs), which was found to be dependent on the constitutive phosphorylation of STAT3 in CSCs 51. Although CSCs have been found to overexpress PD-L1 in a variety of tumors 52, no relevant studies have focused on PD-L1 and CSCs in HCC. Recently, two types of anti‐PD‐1 monoclonal antibodies, nivolumab and pembrolizumab, have been FDA-approved as second‐line therapies for advanced HCC, and a small percentage of patients have achieved complete remission (CR), resulting in long-term survival 53, 54. Therefore, we speculate that anti-PD-1 therapy may be effective in clearing PD-L1-overexpressing CSCs in CR patients with HCC, which needs to be validated in future studies.

## External mechanisms of immune evasion

### HBV/HCV infection

HBV and HCV infections are major risk factors for HCC development and are also associated with the acquisition of a stem-like phenotype in HCC 55-58. Hepatitis B virus X protein (HBx) is a 16.5 KDa protein, which has been shown to promote the expression of hepatoma stem cell markers (including EpCAM, CD133, CD90, *etc*.), contributing to tumor initiation and migration 59, 60. In addition, chronic HCV infection can potentiate CSC generation by inducing CaM kinase-like-1 (DCAMKL-1), EMT, and hepatic stem cell-related factors 55, 56. Moreover, chronic HBV/HCV infection promoted a viral-related inflammatory environment, which increased the expression of stemness-related properties (OCT4/Nanog, IGF-IR) by inflammatory cytokines in HCC 61. Chang and colleagues also demonstrated that the activation of IL6/IGFIR through induction of OCT4/NANOG expression was related to poor prognosis in HBV-related HCC 62. Furthermore, a virus-associated inflammatory microenvironment can antagonize the antiviral immune response, as well as the antitumor response through recruitment of macrophages and the secretion of IL-6 61, 62.

### Alcoholic and nonalcoholic steatohepatitis

As we known, alcoholic, and nonalcoholic steatohepatitis (NASH) have emerged as an important risk factor in the development of HCC 63. Chronic alcohol intake favors the formation of chronic inflammation, which induces reactive oxygen species (ROS) and DNA damage, thereby facilitating the activation of mutations in tumor stem cell-associated genes. Several studies have demonstrated that alcohol can induce the emergence of CSCs in HCC 64. Machida and colleagues suggested that Toll-like receptor 4 (TLR4) plays an important role in the induction of synergistic liver oncogenesis by alcohol and HCV, depending on an obligatory function for Nanog, a stem cell marker of TLR4 downstream gene 65. In addition, CD133^+^/CD49f^+^ tumor stem cells isolated from alcohol-fed HCV Ns5a or core transgenic mice, are tumorigenic based on the roles of TLR4 and Nanog, which is correlated with TGF-β signaling pathway due to Nanog-mediated expression of IGF2BP3 and YAP1 66. Ambade *et al*. found that alcoholic steatohepatitis accelerates early HCC by increasing the stemness and miR-122-mediated hypoxia-inducible factor 1α (HIF-1α) activation 67. Related studies also have verified that there is a close link between NASH and CSCs in HCC. Qin and colleagues demonstrated that neuroblastoma derived homolog (MYCN) high expression (MYCN^high^) CSC-like HCC cells have more unsaturated fatty acids, and lipid desaturation-mediated endoplasmic reticulum (ER) stress signaling regulates the expression of MYCN gene in HCC CSCs 68. Chong *et al*. showed that saturated fatty acid can induce the properties of CSC in HCC through NF-κB activation 69. Moreover, NASH can lead to the reshaping of local TME, which weakens the antitumor functions of CD4^+^ T cells, cytotoxic CD8^+^ T cells, natural killer (NK) cells and Th17 cells 70-74. Additionally, alcohol or NASH-related HCC usually develops with advanced liver fibrosis and cirrhosis, which can induce the formation of hypoxia, contributing to CSC-mediated immune escape in HCC.

### Hypoxia and Angiogenesis

Hypoxia is common in HCC, especially in patients with liver cirrhosis 75. Hypoxia can induce EMT and increase the expression of stemness-related genes, which further increases the proportion of CSCs in HCC 76-79. HIF-1a is a major transcription factor involved in the hypoxic response of hepatoma cells. Ye *et al*. demonstrated that HIF-1a-induced EMT led to the creation of an immunosuppressive TME to promote the metastasis of hepatocellular carcinoma cells. They found that hypoxia-induced EMT of hepatoma cells recruited and educated suppressive indoleamine 2,3-dioxygenase (IDO)-overexpressing TAMs to inhibit T-cell responses and promote immune tolerance in a CCL20-dependent manner 76. Zhang and colleagues found that under a hypoxic microenvironment, the HIF-1a/IL-1b signaling loop between hepatoma cells and TAMs can promote EMT of cancer cells and metastasis 80. Therefore, hypoxia can induce the phenotype of CSCs and further promote a suppressive TME, allowing tumor cells to escape antitumor immunity 81, 82.

An overwhelming number of studies have described the close crosstalk between CSCs and angiogenesis in the TME of various tumors, including HCC 29, 83, 84. VEGF is an important pro-angiogenic factor that has been shown to play a key role in the generation of a pro-angiogenic TME. Tang and colleagues documented that CD133^+^ CSCs of HCC can promote tumor angiogenesis through neurotensin/interleukin-8/CXCL1 signaling. Moreover, HCC CSCs preferentially secrete exosomes to promote VEGF secretion from endothelial cells, which in turn promotes tumor angiogenesis 85. Liu *et al*. found that VEGF increases the proportion of CD133^+^ CSCs by activating VEGFR2 and enhances their self-renewal capacity by inducing Nanog expression in HCC 86. Meanwhile, VEGF plays an important role in attenuating antitumor effects by negatively affecting antigen-presenting cells (APCs, such as DCs) and effector T cells while positively affecting suppressor immune cells (e.g., TAMs, Tregs, and MDSCs) 87, 88. In sum, the crosstalk between CSCs and angiogenesis may contribute to the suppressive immune microenvironment and immune evasion observed in HCC.

Intratumoral hypoxia is a key driver of tumor angiogenesis 89. Related studies have suggested that HIF-1α can bind to the promoter region of the VEGF gene and promote VEGF expression 90. In summary, the close link between hypoxia, CSCs, and angiogenesis may play an important role in antitumor immunity evasion for HCC patients.

### CSC-suppressive immune cell interactions

Over the decades, a large number of studies have been accumulated that extensively describe the interaction of CSCs with the immune system 91, 92. TAMs, as one of the most infiltrating inflammatory cells in the TME, are classified as M1 (tumor-suppressing phenotype) and M2 (tumor-promoting phenotype) macrophages (MΦs). In the TME, TAMs are mostly M2 MΦs that play an important role in attenuating the antitumor immune response 93, 94. Several studies have revealed that CSCs and TAMs can interact closely with each other to suppress antitumor immune effects in various tumors 95, 96. Prostate CSCs can secrete some immunosuppressive molecules, such as TGF-β and IL-4, to promote M2 MΦ polarization 97. CSCs in glioblastoma multiforme can secrete periostin to recruit TAMs 98. Emerging evidence has also revealed that TAMs play a predominant role in the induction and maintenance of CSCs in various tumors by some secretory proteins 96. In HCC, TAM-derived IL-6 can promote the expansion of CD44^+^ CSCs via the STAT3 signaling pathway 99. At the same time, TAMs can also secrete TGF-β to promote CSC-like properties by inducing EMT in HCC 100. As previously described, CD47 has been identified as a marker of CSCs in HCC, which can escape phagocytosis by M1 MΦs in the TME 16, and hypoxia-induced CSCs can secrete CCL20 to recruit IDO^+^ TAMs to inhibit T-cell responses and promote immune tolerance 76. Altogether, these findings indicate a predominant role of TAMs in driving the immune evasion of CSCs in HCC. Moreover, NK cells, as key components of the innate immune system, are anti-tumor effector cells, which also can be impaired by soluble cytokines present in the TME (including CSC-derived cytokines), such as PGE2, IL-10, TGF-β1, granulin-epithelin precursor (GEP), and IDO 101, 102. In HCC, GEP is overexpressed in tumor tissue but not in the adjacent normal tissue, which regulated the expression of CSC-related signaling molecules β-catenin, Nanog, Oct4, and Sox2 103. And, GEP renders hepatocellular carcinoma cells resistant to NK cytotoxicity by down-regulating surface expression of MHC class I chain-related molecule A (MICA), ligand for NK activated receptor NK group 2 member D (NKG2D), and up-regulating human leukocyte antigen-E (HLA-E), ligand for NK inhibitory receptor CD94/NKG2A 102.

MDSCs are another type of suppressive immune cell that seems to enable CSCs to escape antitumor immunity 104. Increasing evidence suggests that MDSCs can secrete inflammatory molecules such as prostaglandin E2 (PGE2), IL-6, and nitric oxide (NO) to foster stemness of tumor cells in cervical cancer or breast cancer 105-107. Conversely, glioblastoma CSCs also promote the survival and immunosuppressive activities of MDSCs by secreting macrophage migration inhibitory factor (MIF) 108. In HCC, MDSCs can inhibit NK cells in patients via the NKp30 receptor 109; hypoxia can induce the recruitment of MDSCs in the TME through chemokine (C-C motif) ligand 26 110. Moreover, Xu *et al*. found that drug-resistant HCC cell-derived IL-6 can enhance the expansion and immunosuppressive function of MDSCs 111. Additionally, HCC CSCs can enhance the production of VEGF, thereby promoting MDSC recruitment in the TME 87. Therefore, the interaction between CSCs and MDSCs can further contribute to immune evasion in HCC. Furthermore, HCC CSCs can attenuate antitumor effects by interacting with Treg cells and cancer-associated fibroblasts (CAFs) 112.

### Immunotherapeutic approaches targeting CSCs

#### Antibody immunotherapy based on markers of CSCs

During the decades, several monoclonal antibodies (mAbs) have been successfully used in clinical patients for the treatment of human cancer, such as antagonists of VEGF, bevacizumab for colorectal cancer, ramucirumab for HCC and so on 113, 114. The mechanisms of antibody-based approaches for targeting CSCs are mainly divided into two parts: the direct inhibitory effect of mAbs and antibody-dependent cellular cytotoxicity (ADCC) 115. Additionally, several bispecific mAbs (BiTE antibodies) consisting of CSC and T cell targets displayed good antitumor effects in some preclinical studies and clinical trials 116.

EpCAM is a common marker of CSCs in HCC. Sun *et al*. indicated that EpCAM^+^ circulating tumor cells were associated with poor prognosis in HCC patients after curative resection 117. In HCC cells, EpCAM expression was found to be dependent the activation of the Wnt/β-catenin signaling pathway, and EpCAM could directly bind to the downstream transcription factor Tcf4, which contributed to the formation of the Tcf4/β-catenin complex 118. In an HCC preclinical study, an EpCAM/CD3 bispecific antibody (anti-EpCAM bispecific T cell engager (BiTE) 1H8/CD3) induced strong peripheral blood mononuclear cell-dependent cellular cytotoxicity, inducing strong elimination of HCC cells *in vitro* and vivo 119. Currently, several II/III stage clinical trials (NCT01320020, NCT00822809, NCT00836654, *etc*.) have shown that BiTE catumaxomab (Anti-EpCAM x Anti-CD3) can effectively improve the quality of life and survival time of malignant ascites (MA) from ovarian and nonovarian (including gastric, pancreatic, and breast, *etc*.) cancer patients 120-122. Moreover, according to the analysis of peritoneal fluid samples from 258 MA patients in a phase II/III study (NCT00836654), catumaxomab therapy can significantly promote the activation of peritoneal T cells and eliminate EpCAM^+^ tumor cells in a manner associated with the release of proinflammatory Th1 cytokines 123. However, in a randomized phase II trial (NCT01504256), compared with chemotherapy alone, catumaxomab followed by chemotherapy did not decrease peritoneal metastasis in gastric cancer patients 124. Therefore, although catumaxomab has achieved promising effects in MA, clinical trials need further exploration in solid tumors, including HCC.

Given that CD47 acts as a marker for HCC CSCs and is crucial for evading phagocytosis by macrophages; thus, targeting CD47 is a promising approach to affect CSCs 125. Preclinical studies demonstrated that anti-CD47 antibody effectively inhibited the growth of HCC, while combination chemotherapy had a synergistic antitumor effect 126, 127. Currently, several anti-CD47 antibodies are currently being studied in clinical trials for a variety of human cancers 128. Related phase I trials suggest that CD47 blockade is well tolerated in patients with hematological malignancies and solid tumors 129, 130. A phase 1b study (NCT02953509) involving patients with relapsed or refractory non-Hodgkin's lymphoma revealed that a total of 50% of the patients had an objective response, with 36% having a complete response, after receiving combination therapy of the Hu5F9-G4 antibody (CD47 blockade) and rituximab 130. However, the effects of antibody-based therapies targeting CD47 need to be further explored in the future in large phase II/III randomized controlled clinical trials.

Based on other HCC CSC markers, such as CD133, CD44, and CD24, related mAbs have demonstrated their effectiveness in eliminating HCC CSCs in preclinical models. Jianhua Huang and colleagues demonstrated that cytokine-induced killer (CIK) cells bound with anti-CD3/anti-CD133 bispecific antibodies can effectively target and kill CD133^+^ HCC CSCs *in vitro* and *in vivo* 131. Wang *et al*. showed that CD44 antibody-targeted liposomal nanoparticles can target and eliminate HCC CSCs in preclinical models 132. In addition, a phase I trial (NCT01358903) involving patients with advanced, CD44-expressing solid tumors revealed that RG7356, an anti-CD44 humanized antibody, is well tolerated but has limited clinical efficacy (21% patients, stable disease) 133. Ma *et al*. suggested that anti-CD24 antibody conjugating doxorubicin can improve antitumor efficacy and has less systemic toxicity in an HCC preclinical model 134. At the same time, a high-affinity humanized anti-CD24 antibody (hG7-BM3-VcMMAE conjugate) was designed to target hepatocellular carcinoma *in vivo* 135. However, these HCC CSC marker-specific, antibody-based therapies require further clinical trials for validation.

### Immune checkpoint inhibitors and antiangiogenic therapy

The rapid development and remarkable success of checkpoint inhibitors in the activation of CTLs led to cancer immunotherapy being named the “Breakthrough of the Year” by Science in 2013 136. Considering that CSCs can induce T-cell apoptosis by high expression of PD-L1, which binds to PD-1, immune checkpoint inhibitors may play an important role in CSC targeted therapy. Two PD-1 inhibitors, nivolumab and pembrolizumab, have been approved by the FDA for HCC after treatment failure on sorafenib based on two phase II trials, the Checkmate-040 study and the Keynote‐224 trial, respectively 137, 138. Reportedly, these two trials demonstrated RECIST1.1 objective response in 15-20% of HCC patients, including a small number of these patients with durable responses.

As one of the most vascular solid tumors, the role of angiogenesis has been extensively studied in HCC, and CSCs play an important role in promoting angiogenesis in HCC. Multiple kinase inhibitors with anti‐angiogenic activity, such as sorafenib and lenvatinib, have been approved by the FDA for the treatment of advanced HCC 139, 140. Additional anti‐angiogenic multi‐kinase inhibitors, such as regorafenib and cabozantinib, have been approved for the treatment of advanced HCC after treatment failure with sorafenib 141, 142. Moreover, according to the results of the phase III trials REACH and REACH‐II, ramucirumab (an anti‐VEGF antibody) has been approved for patients with unresectable HCC with AFP ≥ 400 ng/dL who experience sorafenib failure 143, 144. In sum, these effective antiangiogenic therapies in HCC may exert antitumor effects by indirectly targeting CSCs. In addition, the close crosstalk between CSCs and angiogenesis in the TME of HCC supports an inhibitory immune microenvironment, leading to antitumor immune evasion. Therefore, the combination of immunotherapy with VEGF antagonists in HCC is another new promising direction 145. Recently, a global, open-label, phase III trial (IMbrave150) showed that combining atezolizumab (PD-L1 inhibitor) with bevacizumab resulted in better overall and progression-free survival than sorafenib in patients with unresectable HCC (NCT03434379) 146. Based on these exciting results, the FDA approved bevacizumab in combination with atezolizumab as an updated first-line systemic therapy for patients with unresectable HCC 147. Additionally, the REGONIVO trial (phase Ib trial, NCT03406871) demonstrated that the combination of regorafenib plus nivolumab led to an objective response in 20 patients (40%) with gastric and colorectal cancer 148. Taken together, these findings indicated that checkpoint inhibitors in combination with anti‐angiogenic inhibitors may lead to the depletion of CSCs, which contributed to the success of these trials.

### CAR-T/TCR-T targeting CSCs

The advent of chimeric antigen receptor (CAR) T cell immunotherapy opens a new avenue in adoptive cell therapy, indicating the next breakthrough in immunotherapy 149. According to these unprecedented clinical outcomes of CD19-directed CAR T-cells in patients with certain refractory B cell malignancies, the FDA approved two anti-CD19 CAR-T cell therapies (tisagenlecleucel and axicabtagene ciloleucel) for the treatment of certain hematological malignancies in 2017. Then, the American Society of Clinical Oncology named CAR-T cell therapy “advance of the year” in 2018 150. Indeed, using CAR-T cells to target CSCs is an interesting and promising immunological approach for treating HCC 151. According to the web of clinical trials (https://clinicaltrials.gov/), the most registered type of HCC-related CAR-T clinical trial is GPC-3-targeted CAR-T, mainly because GPC-3 is the specific cell surface marker of HCC 152. In a phase I study (NCT02395250), the results showed that CAR-GPC3 T-cell therapy is well tolerated in GPC3-positive patients with refractory or relapsed HCC, in which two patients had partial responses 153. Moreover, based on the CSC-associated surface markers of HCC, several CAR-T-related clinical trials are ongoing. Wang *et al.* conducted a phase I clinical study (NCT02541370) using autologous CAR-CD133 T-cells to treat 23 patients with advanced and CD133-positive tumors, including 14 advanced HCC patients. The results showed that CAR-CD133 T-cell therapy was feasible and had controllable toxicities; 3 patients achieved partial remission (including 1 HCC patient), and 14 patients (including 9 HCC patients) acquired stable disease; the 3-month disease control rate was 65.2%, and the median progression-free survival was 5 months 154. Additionally, the efficacy of EpCAM-targeted CAR-T cells has been demonstrated preclinically for several solid tumors, such as colon, prostate, and peritoneal cancers 155-158. Currently, one CAR-EpCAM T-cell clinical trial (NCT03013712) has been registered and is recruiting EpCAM-positive cancer (including HCC).

Another promising adoptive cell therapy, T-cell receptor (TCR)-engineered T-cell immunotherapy, has attracted widespread attention and been extensively studied. Compared with CAR-T cells, TCR-T cells can recognize intracellular tumor-associated antigens depending on the MHC complex. In HCC, targeting alpha-fetoprotein (AFP) or HBV/HCV-associated antigens with TCR-T therapies has shown powerful antitumor effects in preclinical models 159-162. Moreover, a series of clinical trials targeting AFP (NCT03971747, NCT04368182) or virus-associated antigens (NCT02686372, NCT03899415) with TCR-T therapies for HCC are currently underway. Considering that HBV and HCV infections contribute to the acquisition of a stem-like phenotype in HCC, TCR-T cells targeting special viral antigens may effectively clear CSCs. In any case, viral antigen-specific TCR-T cell injection may be a promising strategy for HCC.

### NK cell-based cancer immunotherapies

As mentioned previously, CSCs of HCC have low expression of MHC molecules, which contribute to immune escape. Interestingly, the inhibitory receptors of NK cells can recognize MHC-I molecules, hence, NK cells do not usually attack normal cells 163. Therefore, the low-expression of MHC-I on CSCs will make them to be susceptible to be killed by NK cells 164, indicating that NK cell-based cancer immunotherapies may be a promising treatment strategy to target CSCs 32. NK cell-based immunotherapies have achieved encouraging results in hematologic cancers, including IL2-activated haploidentical NK cells infusions 165, and anti-CD19 CAR-NK cell therapy 166, 167. Although some progress is also being made to apply NK cell-based therapies against solid tumors, response rates in patients remain to be unsatisfied 163. In HCC, several NK-cell based I/II phase clinical trials are in progress, such as, autologous/allogeneic NK cells infusion or in combination with other therapies (NCT03319459, NCT04162158, NCT03592706), anti-MUC1 CAR-NK cells (NCT02839954), and so on 168. Moreover, related studies have showed that chemotherapy or radiation therapy can increase the amounts of CSCs in various tumor and induce up-regulating NKG2D ligands MICA and MICB on CSCs, indicating that NK cell-based immunotherapies in combination with radiation therapy or chemotherapy could better eradicate CSCs in HCC 169.

### Vaccines targeting CSCs

CSC-directed immunotherapies to promote tumor cell recognition and elimination by the immune system are mainly focused on the use of DC vaccines 170. Related studies have suggested that DC vaccination using CSC-associated antigens can elicit antigen-specific T-cell responses against CSCs *in vitro* and *in vivo* 171-173. In HCC, Choi and colleagues suggested that DCs stimulated by EpCAM peptides enhance T cell activation and generate CTLs, thus effectively killing HCC CSCs 174. In addition, SP cell lysate-pulsed DCs have been demonstrated to induce a special T cell response against HCC CSCs and suppress tumor growth *in vivo* 172. To date, more than 200 completed clinical trials have involved the use of DC vaccines for cancer treatment. Sipuleucel-T (Provenge) is the only FDA-approved DC vaccine loaded with a fusion antigen protein composed of GM-CSF and prostatic acid phosphatase; it has been used to treat prostate cancer patients and has extended the median overall survival by approximately 4 months 175. Based on the remarkable success of checkpoint inhibitors in the treatment of various tumors in the clinic, DC vaccination in combination with checkpoint inhibitors may be an ideal immunotherapy to foster powerful initial specific effector T cell activation 176. Moreover, as shown in the web of clinical trials (https://clinicaltrials.gov/), a series of clinical trials are ongoing based on DC vaccines (loaded with HCC neoantigens or virus-associated antigens) or combined PD-1 monoclonal antibodies. However, DC vaccination using CSC-associated antigens against HCC needs to be further investigated in future preclinical and clinical trials.

## Conclusions

In summary, accumulating evidence has suggested that CSCs can create an immunosuppressive microenvironment through certain intrinsic and extrinsic mechanisms, resulting in immune evasion in HCC. The intrinsic mechanisms mainly include the following: 1. the activation of immune-related CSC pathways; 2. low-level expression of TAP and/or MHC molecules; and 3. high-level expression of CD47 and PD-L1. The external mechanisms mainly include the following: 1. HBV/HCV infection; 2. alcoholic/nonalcoholic steatohepatitis; 3. hypoxia stimulation; 4. abnormal angiogenesis; and 5. infiltration of suppressive immune cells (**Figure 1**). Currently, immunotherapeutic approaches targeting HCC CSCs mainly include antibody immunotherapy based on CSC markers, immune checkpoint inhibitors, antiangiogenic therapy, CAR-T/TCR-T cell therapy, NK cell-based cancer immunotherapies, and DC vaccines (**Figure 2**).

However, there are still some hindrances to achieving efficacious immunotherapy targeting CSCs in HCC 11, 177. First, the abovementioned immunotherapies targeting CSCs in HCC are based on CSC-specific molecular markers. However, almost all identified stem cell markers are not unequivocally exclusive CSC markers for HCC; in other words, they are also shared with normal stem cells. Second, the existence of intertumor, intratumor, and CSC heterogeneity is a daunting challenge in the development of immunotherapy targeting HCC CSCs 23, 178. Additionally, several studies have demonstrated that the HCC CSCs are plastic and can be converted from tumor cells without a stem phenotype, which can be induced by virus infection, crosstalk between CSCs and tumor cells, hypoxia stimulation, and conventional therapies 76, 77, 106, 179, 180. This plasticity and instability of the CSC phenotype in HCC is a major obstacle for effective immunotherapy targeting CSCs. Considering that CSCs are a rare subpopulation in tumor tissue, targeted CSC therapy alone is presumed to be inadequate for the effective elimination of tumors. Thus, the combination of CSC-targeted immunotherapy with currently used cancer therapies, such as chemotherapy, radiation therapy, antiangiogenic therapy, and checkpoint inhibitors, may effectively eradicate HCC tumors. Moreover, the success of the IMbrave150 trial in advanced HCC patients has illustrated the importance and necessity of combined therapy.

Finally, we think that the most attractive research prospects focused on CSC-targeted immunotherapy in HCC mainly include the following: a) the identification of unequivocal CSC-specific molecular markers through multiomics analyses, such as the combination of proteomics and single-cell analysis; b) dissection of the complex mechanisms of the crosstalk between CSCs and immune cells; and c) validation of the effects of combinatorial treatments in future preclinical and clinical trials, such as DC vaccination (loaded with a mixed CSC and non-CSC special antigens) in combination with checkpoint inhibitors, CAR-T/TCR-T therapy in combination with antiangiogenic therapy, anti-CD47 antibody combined with CAR-T, CAR-T combined NK cell-based cancer immunotherapies, and so on. In conclusion, based on the heterogeneity, plasticity and scarcity of HCC CSCs, it is suggested that combinatorial treatments will be more efficacious than anti-CSC treatment alone. Overall, future immunotherapy should serve as a model for combined therapy.
